# Improving clinical trials using Bayesian adaptive designs: a breast cancer example

**DOI:** 10.1186/s12874-022-01603-y

**Published:** 2022-05-04

**Authors:** Wei Hong, Sue-Anne McLachlan, Melissa Moore, Robert K. Mahar

**Affiliations:** 1 Department of Medical Oncology, St Vincent’s Hospital Melbourne, 41 Victoria Parade, Fitzroy, VIC 3065 Australia; 2 Department of Medicine, St Vincent’s Hospital Melbourne, University of Melbourne, Parkville, VIC Australia; 3 Biostatistics Unit, Centre for Epidemiology and Biostatistics, Melbourne School of Population Health, Faculty of Medicine, Dentistry, and Health Sciences, University of Melbourne, Parkville, VIC Australia; 4 Clinical Epidemiology and Biostatistics Unit, Murdoch Children’s Research Institute, Parkville, VIC Australia

**Keywords:** Bayesian adaptive trial, Time-to-event, Predictive probability of success

## Abstract

**Background:**

To perform virtual re-executions of a breast cancer clinical trial with a time-to-event outcome to demonstrate what would have happened if the trial had used various Bayesian adaptive designs instead.

**Methods:**

We aimed to retrospectively “re-execute” a randomised controlled trial that compared two chemotherapy regimens for women with metastatic breast cancer (ANZ 9311) using Bayesian adaptive designs. We used computer simulations to estimate the power and sample sizes of a large number of different candidate designs and shortlisted designs with the either highest power or the lowest average sample size. Using the real-world data, we explored what would have happened had ANZ 9311 been conducted using these shortlisted designs.

**Results:**

We shortlisted ten adaptive designs that had higher power, lower average sample size, and a lower false positive rate, compared to the original trial design. Adaptive designs that prioritised small sample size reduced the average sample size by up to 37% when there was no clinical effect and by up to 17% at the target clinical effect. Adaptive designs that prioritised high power increased power by up to 5.9 percentage points without a corresponding increase in type I error. The performance of the adaptive designs when applied to the real-world ANZ 9311 data was consistent with the simulations.

**Conclusion:**

The shortlisted Bayesian adaptive designs improved power or lowered the average sample size substantially. When designing new oncology trials, researchers should consider whether a Bayesian adaptive design may be beneficial.

## Introduction

Randomised controlled trials are the gold standard for demonstrating the efficacy of new treatments in evidence-based medicine. Traditional randomised controlled trials recruit up to a fixed sample size that is calculated according to a desired power, significance level, and estimated effect size (we herein refer to such designs as “fixed designs”). Poor a priori understanding about effect sizes can result in under- or over-powered fixed designs. An overpowered trial design will continue to randomise patients to an inferior treatment without good cause. An underpowered trial design will fail to reach a definitive conclusion because the estimated treatment effect is too uncertain. Because a fixed design generally cannot be changed once recruitment is underway without either compromising the integrity of a trial or incurring statistical penalties, even if reliable interim effect sizes become available, the problem of under- or over-powering is real.

Adaptive designs can avoid being under- or over-powered by modifying the design based on interim results. At an interim analysis, an adaptive design can avoid overpowering by stopping the trial if a strong treatment effect is observed, or avoid underpowering by increasing the maximum sample size if it is likely to be too small. Advanced adaptive designs can be very flexible and are often able to investigate multiple treatments in multiple populations simultanously, and reach conclusions earlier than fixed trial designs [1]. Although adaptive designs can use frequentist methods [2–5], Bayesian methods offer more efficiency and flexibility [6].

The advantages of Bayesian adaptive designs are particularly desirable in oncology, where clinical trial failure rates are the highest among all medical specialties [7, 8]. Maximising power for a given sample size is especially important for rare cancers and cancer subtypes, where populations are small.

Despite the potential benefits of Bayesian adaptive designs, they are not widely used [9–11], possibly because the designs are unfamiliar to most researchers and clinicians. Although fixed designs often use simple and well-established methods, there are many different ways to design a Bayesian adaptive trial, with little guidance on what designs are most suitable for a given scenario [12–16]. In contrast to fixed designs where sample sizes are calculated using well-known mathematical formulae, Bayesian adaptive designs generally require computations for which no mathematical formulae exist. Instead, operating characteristics such as type II error (i.e. the false negative rate, noting that 1 less the type II error rate is commonly referred to as the “power”), type I error (i.e. the false positive rate, also commonly referred to as the “significance” level), and average sample size can often only be estimated for adaptive designs by simulating large numbers of virtual trials, requiring specialised software and often considerable computing resources.

Simulations come with simplifications and other assumptions that may not adequately approximate the complexities of reality. “Virtual re-executions,” re-analysing real-world clinical trial data using Bayesian adaptive techniques, provide reassurance that the performance of these designs in simulation translates to the real world, and also serve as case studies that demonstrate the advantages of Bayesian adaptive designs to clinical researchers who may not be familiar with computer simulation methods. Recently, Ryan et al. performed virtual re-executions of a respiratory trial with a binary outcome [17] and an orthopedic trial with a continuous outcome [18] to demonstrate what would have happened if the trials used various Bayesian adaptive designs. We take a similar approach to Ryan et al. [17, 18], but we focus on a medical oncology setting and virtually re-execute a completed phase II breast cancer trial with a time-to-event outcome. Time-to-event outcomes are especially challenging for Bayesian adaptive designs because there is less information to adapt upon at each interim analysis if an event has not yet happened.

We proceed by describing the motivating case study along with shortlisted adaptive designs, then describe the methods to assess the shortlisted designs via simulation and virtual re-execution, follow by describing the results in detail, and conclude with a general discussion and summary of key concepts.

## Methods

### Case study

In 1993, Breast Cancer Trials, known at the time as the Australia and New Zealand Breast Cancer Trials Group, initiated the ANZ 9311 trial [19] to investigate whether increasing dose intensity of chemotherapy improved survival among patients with metastatic breast cancer.

ANZ 9311 was an open-label trial that randomised women with metastatic breast cancer 1:1 to receive either high-dose epirubicin 150 mg/m^2^ and cyclophosphamide 1500 mg/m^2^ with filgrastim every 3 weeks for 3 cycles (HDEC), or standard dose epirubicin 75 mg/m^2^ and cyclophosphamide 750 mg/m^2^ every 3 weeks for 6 cycles (SDEC). The primary outcome was overall survival. A total of 225 participants were deemed sufficient to detect, with 80% power at the 5% significance level, a change in median overall survival of 10 versus 15 months.

The trial recruited 235 participants over the period from April 1994 to July 1998 for an average rate of 4.6 per month. At the time of final analysis, 19.25 years after recruitment began, four participants (1.7%) were lost to follow-up. Median overall survival was 14.5 months in the HDEC arm versus 16.5 months in the SDEC arm (logrank $p = 0.29$).

### Shortlisted fixed and adaptive designs

As a benchmark to compare the Bayesian adaptive designs against, we considered how we would address the ANZ 9311 clinical question today using a fixed design with a shorter follow-up time than the original 19.25 years. The sample size for the fixed design was recalculated using the Kim and Tsiatis [20] method with Freedman’s formula [21]. Survival times were assumed to be exponentially distributed, with estimated median overall survival of 10 months in the control arm and 15 months in the experimental arm. It was assumed that participants would be recruited at an average rate of 4.5 per month, and had a 1% chance of being lost to follow-up per year. Assuming a maximum of 10 years for recruitment and follow-up, 234 participants (117 per arm), yielding 197 events, provided 80% power to detect a hazard ratio corresponding to the estimated survival difference at the 5% two-sided significance level. Hence, the fixed design trial would close to recruitment when 234 participants have been recruited, and final analysis would occur at 197 events.

We shortlisted several adaptive designs for the virtual re-execution of ANZ 9311. Each design specified an interim analysis every 28 days, at which point a probability of efficacy was computed using one of several methods. In general, at each of these interim analyses, the trial stopped for futility if this probability dropped below a lower decision threshold, ${d}_{\mathrm{L}}$, provided at least a minimum number of events for futility, ${k}_{\mathrm{F}}$, had occurred in each arm. The trial stopped for success if this probability exceeded an upper decision threshold, ${d}_{\mathrm{U}}$, provided at least a minimum number of events for success, ${k}_{\mathrm{S}}$, had occurred in each arm. If neither threshold was reached, the trial stopped inconclusively when the maximum sample size was reached in either arm. All adaptive designs that we considered used equal randomisation. The maximum sample size per arm was set at 117 so that it could be compared directly to the fixed design. Several specific decision methods for time-to-event outcomes were considered:

1. Posterior probability: The posterior probability that the mean survival time in the experimental arm is larger than that in the control arm, calculated using the “exponential-inverse gamma model” as described by Thall et al [12].
2. Predictive probability of success (PPS): PPS is the probability that the trial *will stop* for success by the time the maximum sample size is reached, based on data up to and including the current interim. Tang [13] describes a method of calculating the PPS of the Cox proportional-hazards model, where “success” is the two-sided p-value being less than a significance threshold, ${d}_{\mathrm{\alpha }}$, in favour of the experimental arm.
3. Conditional probability of success (CPS): CPS is the probability of observing success given the treatment effect equals a *specific value* (in contrast to PPS, which considers the *entire posterior probability distribution* of the treatment effect). Because the treatment effect has a range of possible values, so does CPS. Here we use its median value as described by Tang [14].
4. Goldilocks: The “Goldilocks” design is a subtle variation of PPS. Here, futility is determined by PPS assuming recruitment continues until maximum sample size is reached, whereas success is determined by PPS assuming recruitment is closed at the current sample size [15]. We calculate these probabilities using Tang’s methods [13].
5. PPBS: PPS can also be calculated where “success” is in terms of the posterior probability, as described above, exceeding a Bayesian success threshold ${d}_{\mathrm{S}}$ [16] (as opposed to a *p*-value being less than a significance threshold). We refer to this as “predictive probability of Bayesian success” (PPBS) to distinguish it from PPS based on *p*-values.

### Software and simulation settings

Using the R and C +  + programming languages [22], we wrote custom software capable of simulating a range of different trial designs. Recruitment times were generated according to a Poisson process. Average recruitment rate, survival times, and dropout rates were simulated according to the assumptions described in the previous section.

### Selection of shortlisted designs

In adaptive trials, different decision methods and event numbers can result in different operating characteristics. For each of the five decision methods, settings for${k}_{\mathrm{F}}$,${k}_{\mathrm{S}}$, ${d}_{\mathrm{L}}$,${d}_{\mathrm{U}}$,${d}_{\alpha }$, and ${d}_{\mathrm{S}}$ that yielded desirable operating characteristics were found through simulation. Operating characteristics were considered desirable if they met both of the following criteria:

1. Probability of success less than that of the fixed design when the null hypothesis is true (median survival of 10 months in both arms), analogous to improved frequentist type I error.
2. Probability of success greater than that of the fixed design when the true effect size is in the region of the estimated effect size (median survival of 14 months to 16 months in the experimental arm versus 10 months in the control arm), analogous to improved frequentist power.

Table 1 lists the possible design settings that were considered for each of the five decision methods. Every possible combination of settings comprised a single design (thus, we explored 19,500 different designs in total), each of which were simulated over a range of effect size scenarios. For each scenario, operating characteristics such as average sample size were calculated by simulating the trial up to 100,000 times, in line with United States Food and Drug Administration recommendations [23]. Among all designs satisfiying the stated criteria, two shortlisted designs were selected for virtual re-execution for each of the five decision methods, one that prioritised low sample size and another that prioritised high power.

**Table 1:** Candidate design settings for each decision method

Parameter	Posterior	PPS, CPS, Goldilocks	PPBS
**Minimum number of events**			
To stop for futility (*k*_F_)	10, 20,..., 50		
To stop for success (*k*_S_)	10, 20,..., 50		
**Decision method thresholds**			
Lower (*d*_L_)	0.05, 0.1, 0.2,..., 0.5	0.005, 0.01, 0.025, 0.05, 0.1, 0.2	
Upper (*d*_U_)	0.99, 0.991,..., 0.999	0.95, 0.975, 0.99, 0.995, 0.999, 0.9995	
Significance (*d*_α_)	–	0.03, 0.04,..., 0.07	–
Bayesian success (*d*_S_)	–	–	0.965, 0.97,..., 0.985

*PPS* Predictive probability of success, *CPS* Conditional probability of success, *PPBS* Predictive probability of Bayesian success

### Virtual re-execution of shortlisted designs

We re-executed each of the shortlisted designs using the real-world data from ANZ 9311. Recruitment times, arm allocations, and clinical outcomes remained as they were in the original trial. At each interim analysis, the Bayesian computations were performed using the data that would have been known at the time. The virtual re-execution of a shortlisted design corresponds to what *would have happened* had that design been used for the real-world realisation of the ANZ 9311 trial. As each such re-execution represents only a single realisation of the trial, virtual re-executions were also performed using bootstrapping to estimate the *probability of these results happening* if ANZ 9311 were repeated. Here, participants were still recruited at the original times, but arm allocations were re-randomised and clinical outcomes are randomly sampled with replacement from among the observed outcomes of each respective arm. Bootstrapping was performed using 100,000 replicates. If the list of original recruitment times was exhausted in any virtual re-execution, additional recruitment times were generated according to a Poisson process, with an average recruitment rate equal to that observed for ANZ 9311.

## Results

### Comparison of decision methods

**Fig. 1:**
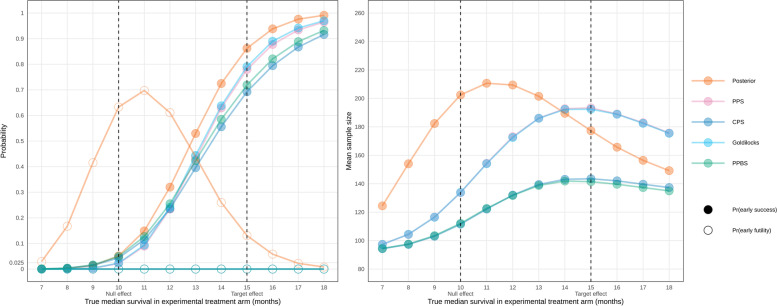
Simulated operating characteristics comparing five different decision methods**.** PPS: predictive probability of success; CPS: conditional probability of success; PPBS: predictive probability of Bayesian success

Figure 1 compares the probabilities of stopping for efficacy and mean sample size for the five trial designs across a range of plausible effect sizes. Each design used a different decision method while keeping the other settings at${k}_{\mathrm{F}}=20$,${k}_{\mathrm{S}}=20$, ${d}_{\mathrm{L}}=0.1$,${d}_{\mathrm{U}}=0.99$, and where applicable,${d}_{\alpha }=0.05$,${d}_{\mathrm{S}}=0.975$. For these settings, the PPS, CPS, Goldilocks, and PPBS designs were much more “decisive” than the posterior probability designs in that they had minimal probability of reaching maximum sample size with an inconclusive result. PPS and Goldilocks performed similarly to each other, with Goldilocks having a very small advantage over PPS in terms of higher power and lower mean sample size. CPS and PPBS also performed similar to each other, with both yielding lower power and lower mean sample size compared to PPS and Goldilocks.

### Shortlisted designs and operating characteristics

We were able to find designs that satisfied our selection criteria for each of the five decision methods. Table 2 describes the ten shortlisted designs that best prioritised either low sample size or high power. Table 3 describes the operating characteristics of each design in terms of type I and II errors and the sample size distribution at both the null and the target effect. Adaptive designs that prioritised small sample size reduced average sample size by up to 37% when there was no clinical effect and by up to 17% at the target clinical effect while still achieving desirable type I and II errors. Adaptive designs that prioritised high power yielded an absolute increase in power by up to 5.9% while still achieving desirable type I errors. Among the candidate designs, PPS and Goldilocks achieved the smallest average sample sizes, whereas PPS, Goldilocks and PPBS achieved the highest power.

**Table 2:** Settings for each shortlisted design

Strategy	Parameter	Posterior	PPS	CPS	Goldilocks	PPBS
Small average sample size	*k*_F_	20	10	30	10	30
	*k*_S_	50	10	40	20	50
	*d*_L_	0.2	0.025	0.01	0.025	0.01
	*d*_U_	0.993	0.99	0.999	0.975	0.975
	*d*_α_	–	0.04	0.05	0.04	–
	*d*_S_	–	–	–	–	0.98
High power	*k*_F_	30	50	50	50	50
	*k*_S_	50	10	50	50	50
	*d*_L_	0.05	0.01	0.01	0.01	0.005
	*d*_U_	0.993	0.9995	0.9995	0.995	0.9995
	*d*_α_	–	0.05	0.05	0.05	–
	*d*_S_	–	–	–	–	0.975

*PPS* Predictive probability of success, *CPS* Conditional probability of success, *PPBS* Predictive probability of Bayesian success

**Table 3:** Simulated operating characteristics for shortlisted designs

Design	Type I error (%)	Power (%)	Sample size, mean (SD)	
			**Null effect**	**Target effect**
**Fixed**	2.50	80.8	235 (1.9)	235 (1.9)
**Small sample size**				
Posterior	2.46	81.3	184 (59.8)	211 (25.3)
PPS	2.26	81.4	149 (59.2)	199 (50.4)
CPS	2.39	81.8	154 (44.5)	207 (34.8)
Goldilocks	2.45	81.7	149 (59.0)	194 (47.6)
PPBS	2.42	81.5	153 (43.3)	205 (29.5)
**High power**				
Posterior	2.45	82.1	223 (30.5)	213 (20.7)
PPS	2.44	85.9	198 (29.1)	225 (25.4)
CPS	2.36	84.8	187 (28.2)	222 (19.2)
Goldilocks	2.42	85.9	198 (29.0)	224 (17.4)
PPBS	2.44	85.9	191 (29.4)	223 (18.6)

*PPS* Predictive probability of success, *CPS* Conditional probability of success, *PPBS* Predictive probability of Bayesian success. The target effect represents the scenario of a true median survival of 15 months in the experimental arm and 10 months in the control arm

**Fig. 2:**
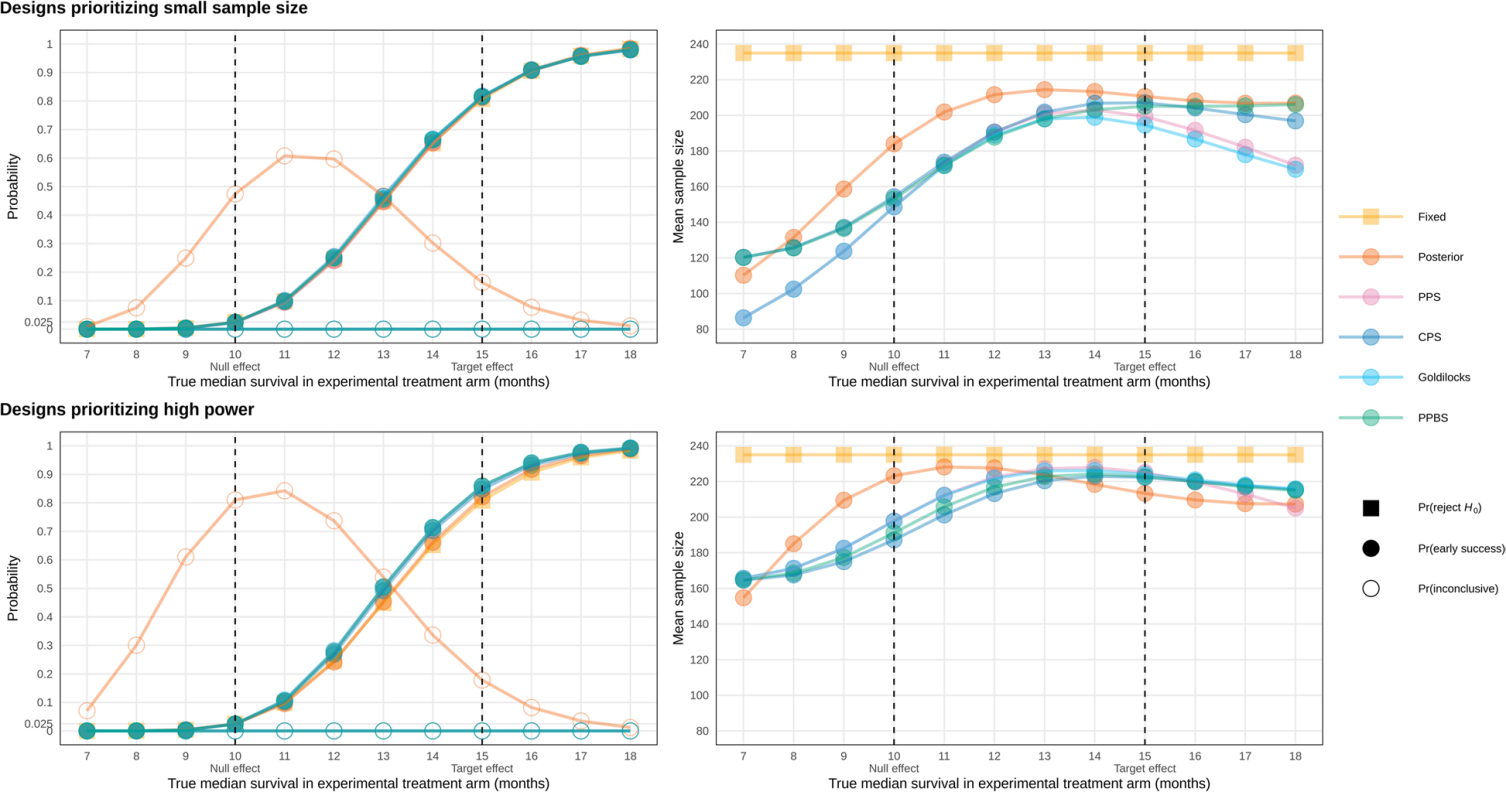
Simulated operating characteristics of shortlisted designs. PPS: predictive probability of success; CPS: conditional probability of success; PPBS: predictive probability of Bayesian success

Figure 2 illustrates the operating characteristics of the ten shortlisted designs across the range of plausible effect sizes.

### Virtual re-execution of ANZ 9311

Table 4 describes what would have happened had each shortlisted design been used for ANZ 9311. All but one of the shortlisted adaptive designs stopped early for futility, arriving at the equivalent conclusion as the original trial, but doing so while reducing sample size by up to 33% compared to the original trial. To address the question of whether these benefits would be *consistently* realised if ANZ 9311 were to be repeated by recruiting a new sample from the same population, Table 5 describes the estimated operating characteristics of each shortlisted design using bootstrapped ANZ 9311 data. The demonstrated behaviour of shortlisted designs during virtual re-execution was consistent with their simulated operating characteristics. All shortlisted designs had a very low probability of stopping for success, of around 0.1%. Posterior probability designs had a substantial chance of reaching maximum sample size without a conclusion, but PPS, CPS, Goldilocks and PPBS almost always stopped early for futility. When prioritising small sample size, PPS and Goldilocks yielded the largest improvement compared to the fixed design, reducing average sample size by 42%. Shortlisted designs prioritising high power, despite having the highest tendency to recruit more participants in pursuit of this goal, still reduced average sample size by 6–9% compared to the fixed design.

**Table 4:** Virtual re-execution of shortlisted designs using real-world ANZ 9311 data

Design	Trial conclusion	Duration (months)	Sample size	Hazard ratio (95% CI)
**Original trial**	*H*_0_ not rejected	53.0 recruitment + 91.2 follow-up	235	1.17 (0.90, 1.52)
**Small sample size**				
Posterior	Early futility	69.9	233	1.14 (0.85, 1.52)
PPS	Early futility	50.6	214	1.03 (0.71, 1.48)
CPS	Early futility	43.2	164	1.00 (0.66, 1.52)
Goldilocks	Early futility	50.6	214	1.03 (0.71, 1.48)
PPBS	Early futility	42.3	158	0.98 (0.64, 1.50)
**High power**				
Posterior	Inconclusive	120.5	233	1.17 (0.90, 1.53)
PPS	Early futility	52.4	228	1.10 (0.77, 1.58)
CPS	Early futility	47.8	189	1.00 (0.68, 1.47)
Goldilocks	Early futility	52.4	228	1.10 (0.77, 1.58)
PPBS	Early futility	47.8	189	1.00 (0.68, 1.47)

*PPS* Predictive probability of success, *CPS* Conditional probability of success, *PPBS* Predictive probability of Bayesian success. The hazard ratios and 95% confidence intervals (CI) are the estimates from the Cox proportional-hazards model, unadjusted for any adaptations, for illustrative purposes only

**Table 5:** Virtual re-execution of shortlisted designs using bootstrapped ANZ 9311 data

Design	Duration (months), mean (SD)	Sample size, mean (SD)	Probability of success (%)	Probability of inconclusive (%)	Hazard ratio, median (IQR)
**Fixed**	76.7 (3.93)	233 (0.0)	0.13	–	1.16 (1.06, 1.28)
**Small sample size**					
Posterior	55.2 (32.3)	164 (51.3)	0.10	15.7	1.27 (1.15, 1.43)
PPS	36.9 (13.1)	134 (51.8)	0.13	0.05	1.32 (1.10, 1.62)
CPS	42.3 (9.7)	154 (28.9)	0.07	0.09	1.15 (1.03, 1.36)
Goldilocks	36.9 (13.1)	134 (51.8)	0.14	0.05	1.32 (1.10, 1.62)
PPBS	41.0 (5.6)	152 (26.6)	0.12	0.00	1.15 (1.02, 1.36)
**High power**					
Posterior	88.1 (35.4)	214 (32.5)	0.10	48.5	1.22 (1.07, 1.43)
PPS	53.6 (9.2)	218 (14.6)	0.06	0.10	1.17 (1.07, 1.33)
CPS	52.0 (8.1)	213 (15.1)	0.05	0.09	1.17 (1.03, 1.33)
Goldilocks	53.5 (9.2)	218 (14.6)	0.06	0.09	1.17 (1.07, 1.33)
PPBS	50.9 (4.5)	214 (15.1)	0.18	0.00	1.17 (1.03, 1.33)

*PPS* Predictive probability of success, *CPS* Conditional probability of success, *PPBS* Predictive probability of Bayesian success. The median and interquartile range (IQR) of the distribution of 100,000 bootstrapped hazard ratios are shown, unadjusted for any adaptations, for illustrative purposes only

## Discussion

We have shown how Bayesian adaptive designs can substantially outperform a fixed design in terms of lower type I and II errors, and average sample size in a real-world oncology clinical trial. The virtual re-executions of real-world data were consistent with the simulation results. Although in general delayed outcomes are problematic for adaptive trial designs [24], we were able to demonstrate the benefits of using such designs across a range of median survival times in the range of 7 to 18 months via simulation. The shortlisted designs are specific to our clinical scenario; other clinical scenarios would require different designs to be found through their own simulations [25]. In particular, it may be harder to find adaptive designs with good operating characteristics in scenarios with longer survival times, faster recruitment rates, and more loss to follow-up. Note also that the posterior probability and PPBS designs assumed exponentially distributed survival times which may be not be suitable for studies where this assumption is unjustifiable.

Our study considered five different decision methods for early stopping. To our knowledge, this is the first time these have been directly compared in this way. For all five decision methods, we were able to shortlist designs with higher power, lower type I error, and lower average sample size compared to the fixed design. Among the five decision methods, the shortlisted designs using PPS, CPS, Goldilocks, and PPBS had noticeably better operating characteristics than those using posterior probability. This may be because trials governed by posterior probability decisions have a substantial probability of reaching maximum sample size without reaching a conclusion, resulting in inefficiency, whereas this is not the case for with PPS, CPS, Goldilocks, and PPBS-based decision methods. Overall, PPS and the closely related Goldilocks decision method offered the best performance, whether prioritising small average sample size or high power. This pattern was consistent across a wide range of simulated effect sizes, and also when the shortlisted designs were applied to real-world trial data. This may be because PPS and Goldilocks are inherently more efficient than the other decision methods, or because our search space of candidate designs happened to be more suited towards them. Whatever the case, PPS and Goldilocks may offer more potential than posterior probability in designs for similar clinical scenarios.

Bayesian adaptive trials have potential to substantially improve the way trials are conducted in medical oncology. Smaller average sample sizes in medical oncology trials would mean better cancer treatments are identified more quickly, and with lower research and development costs, including fewer patients randomised to suboptimal treatments. Higher power means fewer effective treatments remaining unidentified.

To foster the the use of Bayesian adaptive designs their unfamiliarity among researchers must continue to be addressed. Studies such as this one, using real-world trial data in virtual re-executions, are important in addressing such unfamiliarity.

## Conclusion

Compared to fixed designs, Bayesian adaptive designs can offer substantial improvements in terms of higher power and lower average sample, even for time-to-event outcomes with median times in the order of many months. Researchers in oncology designing new studies should consider whether their trials would benefit from using a Bayesian adaptive design.

## Data Availability

The data that support the findings of this study are available from Breast Cancer Trials (formerly known as the Breast Cancer Institute of Australia and the Australia and New Zealand Breast Cancer Trials Group) but restrictions apply to the availability of these data, which were used under license for the current study, and so are not publicly available. Data are however available from the authors upon reasonable request and with permission of Breast Cancer Trials.

## Abbreviations

CPS: Conditional probability of success
HDEC: High-dose epirubicin/cyclophosphamide
PPBS: Predictive probability of Bayesian success
PPS: Predictive probability of success
SDEC: Standard dose epirubicin/cyclophosphamide
